# *ARNTL* (*BMAL1*) and *NPAS2* Gene Variants Contribute to Fertility and Seasonality

**DOI:** 10.1371/journal.pone.0010007

**Published:** 2010-04-02

**Authors:** Leena Kovanen, Sirkku T. Saarikoski, Arpo Aromaa, Jouko Lönnqvist, Timo Partonen

**Affiliations:** 1 Department of Mental Health and Substance Abuse Services, National Institute for Health and Welfare, Helsinki, Finland; 2 Department of Alcohol, Drugs and Addiction, National Institute for Health and Welfare, Helsinki, Finland; 3 Division of Welfare and Health Policies, National Institute for Health and Welfare, Helsinki, Finland; 4 Department of Psychiatry, University of Helsinki, Helsinki, Finland; Leiden University Medical Center, Netherlands

## Abstract

**Background:**

Circadian clocks guide the metabolic, cell-division, sleep-wake, circadian and seasonal cycles. Abnormalities in these clocks may be a health hazard. Circadian clock gene polymorphisms have been linked to sleep, mood and metabolic disorders. Our study aimed to examine polymorphisms in four key circadian clock genes in relation to seasonal variation, reproduction and well-being in a sample that was representative of the general population, aged 30 and over, living in Finland.

**Methodology/Principal Findings:**

Single-nucleotide polymorphisms in the *ARNTL*, *ARNTL2*, *CLOCK* and *NPAS2* genes were genotyped in 511 individuals. 19 variants were analyzed in relation to 31 phenotypes that were assessed in a health interview and examination study. With respect to reproduction, women with *ARNTL* rs2278749 *TT* genotype had more miscarriages and pregnancies, while *NPAS2* rs11673746 *T* carriers had fewer miscarriages. *NPAS2* rs2305160 *A* allele carriers had lower Global Seasonality Scores, a sum score of six items i.e. seasonal variation of sleep length, social activity, mood, weight, appetite and energy level. Furthermore, carriers of *A* allele at *NPAS2* rs6725296 had greater loadings on the metabolic factor (weight and appetite) of the global seasonality score, whereas individuals with *ARNTL* rs6290035 *TT* genotype experienced less seasonal variation of energy level.

**Conclusions/Significance:**

*ARNTL* and *NPAS2* gene variants were associated with reproduction and with seasonal variation. Earlier findings have linked *ARNTL* to infertility in mice, but this is the first time when any polymorphism of these genes is linked to fertility in humans.

## Introduction

Circadian rhythms are the approximate 24-hour oscillations in behavioral or physiological processes that allow organisms to anticipate routine environmental changes and to prepare for the appropriate alignment in order to adapt. Circadian rhythms are generated by the intrinsic clocks whose principal pacemaker is located in the suprachiasmatic nuclei of the anterior hypothalamus. This internal clock is synchronized to the external 24-hour clock by following time-giving cues, primarily the daily light-dark transitions, in the habitat. The principal circadian clock coordinates peripheral oscillators that maintain the timing for a range of physiological functions such as hormone release, body temperature, cardiovascular function and physical activity. Recently, there has been growing interest in the impact of disruption of these rhythms on health.

At the molecular level, circadian rhythms are generated by a network of proteins. The clock protein (CLOCK) [1] pairs up with aryl hydrocarbon receptor nuclear translocator-like (ARNTL or BMAL1) protein [2]. Neuronal PAS domain protein 2 (NPAS2) [3] can substitute for CLOCK and aryl hydrocarbon receptor nuclear translocator-like 2 (ARNTL2 or BMAL2) [4] for ARNTL. Paired CLOCK/NPAS2–ARNTL/ARNTL2 heterodimers thereafter activate the transcription of their target genes [5]-[7].

So far, genetic variations in circadian clock genes have been associated with sleep, mood, and metabolic disorders [8]. Concerning these disorders, *CLOCK* gene variants have been linked to diurnal preference [9], [10], delayed sleep phase syndrome [11], metabolic syndrome and obesity [12], *ARNTL* gene variants to bipolar disorder [13], type 2 diabetes and hypertension [14], and *NPAS2* gene variants to diurnal preference and seasonal affective disorder [15], [16]. However, in some cases the aforementioned associations are conflicting [17], [18], and the established links between circadian clock gene polymorphisms and disease susceptibility therefore remain incomplete.

Our aim was to examine the role of 19 single-nucleotide polymorphisms (SNPs) of four canonical circadian clock genes (*ARNTL*, *ARNTL2*, *CLOCK*, *NPAS2*) in relation to a range of health-related phenotypes that were assessed with structured methods as part of a population-based health interview and examination study. Here, we report associations with seasonal variation, reproduction, and social activity.

## Methods

### Subjects

This study was part of a nationwide health interview and examination survey whose design and methods are described in detail on the http://www.terveys2000.fi/indexe.html site. The ethical approval of the survey has been accepted by the ethics committee of the National Public Health Institute and all participants provided written informed consent. Of the 7415 participants aged 30 or over, 5480 took part to a health status examination and a diagnostic mental health interview (M-CIDI [19]), a valid and reliable instrument for the assessment of alcohol use, mood and anxiety disorders, yielding the diagnosis according to DSM-IV, filled in the self-report questionnaires, and gave a venous blood sample for DNA extraction. The sample that was drawn from the study for our analysis included 511 individuals (9.3% of the eligible participants) having no diagnosis of mental disorder. Of the study subjects that were originally selected as healthy controls for those with alcohol use disorder, 412 were men and 99 women.

### Analyzed phenotypes

The study sample that was derived from an epidemiological nation-wide cohort, representative of the population, is well characterized. This allowed an extensive phenotype analysis concerning data on reproduction, seasonal variation and mental well-being. Altogether 31 phenotypes were analyzed (Table 1).

**Table 1:** Phenotypes analyzed in the study.^a^

Phenotype	A	B
Reproduction		
Men: Infertility	35	376
Women: Infertility	19	80
Irregular or no periods in women less than 54 years old	20	61
Number of pregnancies	99	
Number of miscarriages	85	
High blood pressure during pregnancy	23	59
Seasonal variation		
Global seasonality score (GSS)	511	
Global seasonality score factor 1 (GSS1)	511	
Global seasonality score factor 2 (GSS2)	511	
Seasonal variation in sleep length	364	147
Seasonal variation in social activity	348	163
Seasonal variation in mood	364	147
Seasonal variation in weight	225	286
Seasonal variation in appetite	187	324
Seasonal variation in energy	362	149
If seasonal variation experienced in above, a problem?	40	391
Vitamin D, S-D-25, nmol/l	504	
Well-being		
Beck Depression Inventory (BDI)	510	
Beck Depression Inventory factor 1 (BDI1)	499	
Beck Depression Inventory factor 2 (BDI2)	499	
General Health Questionnaire (GHQ)	510	
General Health Questionnaire factor 1 (GHQ1)	500	
General Health Questionnaire factor 2 (GHQ2)	500	
Health related quality of life factor 1 (15-D 1)	510	
Health related quality of life factor 1 (15-D 2)	510	
Health related quality of life factor 1 (15-D 3)	510	
Epworth Sleepiness Scale factor 1 (ESS1)	490	
Epworth Sleepiness Scale factor 2 (ESS2)	490	
Hours of sleep in 24 hours	500	
Social activity	194	119
Maslach Burnout Inventory (MBI)	396	

a Positive (A) and negative (B) status with respect to the phenotype analyzed. In case of continuous variables, the number of subjects is given in the column A.

Regarding reproduction, the menstrual cycle (irregular or no periods versus regular periods in women aged 54 or under), the number of pregnancies (ended in a delivery, a miscarriage, or an abortion), elevated blood pressure during a pregnancy, the number of miscarriages, and infertility (failure to get pregnant within 12 months) were analyzed. Sample size for these phenotypes was small due to the small number of women in our study.

With regard to seasonal variation in mood and behavior, the participants filled in a modified Seasonal Pattern Assessment Questionnaire (SPAQ) [20]. Seasonal variation in sleep length, social activity, mood, weight, appetite, and energy level were scored from 0 to 3 (none, slight, moderate or marked change) instead of 0 to 4 (none, slight, moderate, marked or extremely marked change). The sum score of the six items is the global seasonality score (GSS). The psychometric properties of this modified questionnaire have been tested [21]. The factor analysis produced two factors: factor one (GSS1) including the items of weight, and appetite; and factor two (GSS2) including the items of sleep length, social activity, mood, and energy level. The participants who reported of having any variation were divided into those who did not experience the variations as a problem and those who experienced them as mild (n = 37), moderate (n = 2), marked (n = 0) and severe (n = 1) problems.

Social activity was assessed with the sum of two questions asking for the frequencies of social contacts at home and those at visit. Concerning mental well-being, the phenotypes analyzed are listed in Table 1 and described in more detail in the supporting information (see Text S1).

### SNP selection and genotyping

Five SNPs for *ARNTL*, *ARNTL2* and *NPAS2* and four SNPs for *CLOCK* were selected (Table 2). SNPs included Hap-Map tag-SNPs and candidate SNPs selected on the basis of previous association studies with health-related condition or behavior.

**Table 2:** Genotype and allele frequencies and Hardy-Weinberg equilibrium estimate P-values.

		Alleles		Genotype counts (%)				Allele counts (%)	
Gene	SNP	1	2	11	12	22	HWE^P^	1	2
*NPAS2*	rs10206927	*G*	*C*	347 (67.9)	150 (29.4)	14 (2.7)	0.76	844 (82.6)	178 (17.4)
*NPAS2*	rs6725296	*G*	*A*	390 (76.3)	116 (22.7)	5 (1.0)	0.31	896 (87.7)	126 (12.3)
*NPAS2*	rs11673746	*C*	*T*	249 (48.7)	208 (40.7)	54 (10.6)	0.3	706 (69.1)	316 (30.9)
*NPAS2*	rs11541353	*C*	*T*	352 (68.9)	143 (28.0)	16 (3.1)	0.76	847 (82.9)	175 (17.1)
*NPAS2*	rs2305160	*G*	*A*	252 (49.3)	218 (42.7)	41 (8.0)	0.59	722 (70.6)	300 (29.4)
*CLOCK*	rs2412646	*C*	*T*	278 (54.4)	197 (38.6)	36 (7.0)	0.91	753 (73.7)	269 (26.3)
*CLOCK*	rs11240	*C*	*G*	221 (43.2)	242 (47.4)	48 (9.4)	0.13	684 (66.9)	338 (33.1)
*CLOCK*	rs2412648	*T*	*G*	208 (40.7)	232 (45.4)	71 (13.9)	0.63	648 (63.4)	374 (36.6)
*CLOCK*	rs3805151	*T*	*C*	182 (35.6)	244 (47.7)	85 (16.6)	0.85	608 (59.5)	414 (40.5)
*ARNTL*	rs6486120	*G*	*T*	277 (54.2)	183 (35.8)	51 (10.0)	0.015	737 (72.1)	285 (27.9)
*ARNTL*	rs1982350	*G*	*A*	179 (35.0)	224 (43.8)	108 (21.1)	0.019	582 (56.9)	440 (43.1)
*ARNTL*	rs3816360	*C*	*T*	151 (29.5)	245 (47.9)	115 (22.5)	0.42	547 (53.5)	475 (46.5)
*ARNTL*	rs2278749	*C*	*T*	327 (64.0)	162 (31.7)	22 (4.3)	0.78	816 (79.8)	206 (20.2)
*ARNTL*	rs2290035	*A*	*T*	175 (34.2)	242 (47.4)	94 (18.4)	0.53	592 (57.9)	430 (42.1)
*ARNTL2*	rs7958822	*G*	*A*	145 (28.4)	262 (51.3)	104 (20.4)	0.53	552 (54)	470 (46)
*ARNTL2*	rs4964057	*T*	*G*	177 (34.6)	241 (47.2)	93 (18.2)	0.52	595 (58.2)	427 (41.8)
*ARNTL2*	rs1037921	*A*	*G*	429 (84.0)	79 (15.5)	3 (0.6)	1	937 (91.7)	85 (8.3)
*ARNTL2*	rs2306074	*T*	*C*	210 (41.1)	240 (47)	61 (11.9)	0.63	660 (64.6)	362 (35.4)
*ARNTL2*	rs35878285	*A*	*-*	(100)				(100)	

Abbreviation: HWE^P^, Hardy-Weinberg equilibrium p-value.

Genomic DNA was isolated from whole blood according to standard procedures. SNPs were genotyped in 10 µl reactions with a fluorogenic 5′ nuclease assay method (TaqMan™) with pre-designed primer-probe kits using Applied Biosystems 7300 Real Time PCR System according to manufacturers' instructions. All the samples were successfully genotyped, and those 5% of the samples that were re-genotyped did confirm the genotyping results to have no error. Genotyping of the first 276 samples indicated that *ARNTL2* rs35878285 was not polymorphic in our sample, and it was therefore excluded from further analysis, leaving 18 SNPs for the statistical analysis.

### Statistical analysis

Statistical analyses of the data were performed by using the PLINK software, version 1.05 (http://pngu.mgh.harvard.edu/purcell/plink/) [22]. The genotype and allele frequencies and the Hardy-Weinberg equilibrium (HWE) estimates were calculated. SNPs were compared between participants with a condition and those without the condition, or in relation to a quantitative measure. Additive, dominant and recessive models were calculated using linear and logistic regression models for the quantitative and categorized traits respectively. Age and sex were controlled for in the analyses. To correct SNP analyses for multiple testing across all the tests performed, the R software (http://www.r-project.org/) was used to calculate false discovery rate (FDR) q-values [23]. The Haploview software [24] was used to calculate the linkage disequilibrium (LD) estimates. The formed haplotype blocks were analyzed by using the PLINK software, with the sliding window approach, regression models, and controlling for age and sex.

## Results

The calculated genotype and allele frequencies and HWE estimates are given in Table 2. All the SNPs were in HWE (P>0.01) in the 511 subjects. The most significant associations (q<0.4) are listed in Table 3, and they are discussed below (see Table S1, for all the associations having P<0.01). One of these associations had a false discovery rate q-value under 0.05. It is of note that there was no significant association with *ARNTL2*. In the haplotype analysis (see Table S2), P-values for haplotypes in the formed blocks (see Figure S1) were higher than for the individual SNPs within the haplotype and thus are not discussed.

**Table 3:** SNPs showing evidence for association at q<0.4.^a^

Variable	Gene	SNP	Test	Re	Beta	S.E.	L95% CI	U95% CI	P-value	q-value
Number of pregnancies	*ARNTL*	rs2278749	REC	*TT*	7.2	2.12	3.04	11.35	0.001	0.28
Number of miscarriages	*ARNTL*	rs2278749	REC	*TT*	4.65	0.83	3.03	6.27	3×10^−7^	0.0004
Number of miscarriages	*NPAS2*	rs11673746	DOM	*T+*	−0.64	0.2	−1.03	−0.25	0.002	0.33
GSS	*NPAS2*	rs2305160	DOM	*A+*	−0.81	0.24	−1.29	−0.33	0.0009	0.28
GSS factor 1	*NPAS2*	rs6725296	ADD	*A*	0.27	0.08	0.11	0.42	0.0008	0.28
GSS factor 1	*NPAS2*	rs6725296	DOM	*A+*	0.27	0.08	0.1	0.43	0.001	0.33

Variable	*Gene*	SNP	Test	Re	OR	S.E.	L95% CI	U95% CI	P-value	q-value
Seasonal variation in weight	*NPAS2*	rs6725296	ADD	*A*	1.95	0.2	1.32	2.89	0.0009	0.28
Seasonal variation in weight	*NPAS2*	rs6725296	DOM	*A+*	1.95	0.21	1.29	2.96	0.002	0.33
Seasonal variation in energy level	*ARNTL*	rs2290035	REC	*TT*	0.45	0.24	0.28	0.72	0.0008	0.28
Social activity	*CLOCK*	rs2412646	REC	*TT*	0.2	0.53	0.07	0.56	0.002	0.38

a Results presented for the rare allele/genotype.

Abbreviations: GSS, Global Seasonality Score; SNP, single-nucleotide polymorphism; REC, recessive model; DOM, dominant model; ADD, additive model; Re, reference allele/genotype(s); OR, odds ratio; S.E., standard error; L95% CI, lower bound of the 95% confidence interval; U95% CI, upper bound of the 95% confidence interval.

The *A^+^* allelic status (*AA* and *AG* genotypes) of *NPAS2* rs2305160 was over-represented among individuals not experiencing seasonal variation (P<0.001) as measured with the GSS. The *A* allele (P<0.001) and the *A^+^* (*AA* or *AG* genotypes) allelic status (P<0.01) of *NPAS2* rs6725296 were associated with the GSS1 and with the single item of seasonal variation in weight. In addition, people with the *TT* genotype of *ARNTL* rs2290035 were more likely not to experience seasonal variation in energy level (P<0.001).

The *TT* genotype of *ARNTL* rs2278749 was associated with both a higher number of pregnancies (P<0.01) and a higher number of miscarriages (P<0.000001), whereas the *T^+^* (*TT* or *CT* genotypes) allelic status of *NPAS2* rs11673746 was linked to a lower number of miscarriages (P<0.01).

Individuals with the *TT* genotype of *CLOCK* rs2412646 were more likely to have a lower level of social activity (P<0.01).

## Discussion

Our aim was to investigate genetic variants of the four key circadian clock genes *ARNTL*, *ARNTL2*, *CLOCK* and *NPAS2* in relation to a range of health related phenotypes assessed in a representative nation-wide sample of the population aged 30 or over. These genes together with other circadian clock genes generate the circadian rhythms and the seasonal cycles, such as reproduction related activities, as well as have control for the cell-division cycle and the metabolic cycle [25], [26].

Circadian rhythms and clock genes have previously been connected to reproductive functions in animal models [27], but no studies on the role of these genes in human fertility have been published. In studies with *ARNTL* knockout mice [28], both male and female homozygous knockout mice were infertile or had reduced fertility [29]–[32]. Their results indicated an inability to carry on viable pregnancies and some degree of embryo lethality in these mice. The reason for this infertility remains unclear, but Alvarez *et al.* (2008) speculated that it was most likely to result from altered levels of reproductive hormones. We found that the *TT* genotype of *ARNTL* rs2278749 was linked to a higher number of pregnancies and a higher number of miscarriages, whereas and the *T^+^* allelic status of *NPAS2* rs11673746 was linked to a lower number of miscarriages. It may be that *NPAS2* plays a role in the human fertility through its potential effect on appetite and weight that follow a seasonal pattern. All in all, our data now suggest that *ARNTL* and *NPAS2* may be involved also in the human fertility.

We investigated seasonal variation by using Global Seasonality Score, a sum score of six items, i.e. seasonal variation of sleep length, social activity, mood, weight, appetite, and energy level. High global seasonality scores can be indicative of seasonal affective disorder, also known as winter depression that is characterized by routine fluctuations in behavior over the year and depressive episodes during a particular period of the year [33]. Depressive and bipolar disorders as well as their subtypes with the seasonal pattern have circadian rhythm misalignments [34], [35]. In our current study, individuals with the *A^+^* allelic status of *NPAS2* rs2305160 (Ala394Thr) were demonstrated to have lower global seasonality scores indicative of less seasonal variation. Earlier, a haplotype with the *G* allele of rs2305160 was suggestively protective against depression [36]. In the haplotype, rs1374324 was the only single SNP associated with depression and linkage of this SNP to rs2305160 might explain the discrepancy. In our study rs1374324 was not analyzed. The *A^+^* allelic status has previously been associated with lower levels of free and bioavailable testosterone [37]. Testosterone levels exhibit seasonal variation [38]–[40], and the hormone affects both mood and behavior [41]. Here, we thus hypothesize that the effect of *NPAS2* rs2305160 on seasonality may, at least in part, be mediated via an influence on testosterone levels.

*NPAS2* rs2305160 is in LD with *NPAS2* rs11541353 (S471L) that has been demonstrated to associate with hypertension as part of the metabolic syndrome [42]. Here, our results demonstrate that the *A^+^* allelic status of *NPAS2* rs6725296 was associated with loadings on the metabolic factor of the global seasonality score, which is composed of seasonal variation of weight and appetite. The role of NPAS2 in eating behavior is supported by an observation that NPAS2 deficient mice have impaired ability to adapt to restricted feeding schedule [43]. As the circadian, metabolic and cell-division cycles appear to be coordinated in a similar way, deficient NPAS2 may have an adverse effect on these functions [25], [44], [45]. On the other hand, experienced poor lighting increases the risk of the metabolic syndrome through its contribution to the metabolic factor of the global seasonality score [46]. Our findings now suggest that seasonal weight gain bridges *NPAS2* and hypertension to the pathogenesis of the metabolic syndrome. Exposures to light or lighting conditions may modify the pathogenesis through their actions on testosterone production [47] and its subsequent effects. Here, no significant association was observed for *NPAS2* rs11541353 even though this SNP has been linked to seasonal affective disorder [15], the association however being only with the clinical diagnosis but not with the GSS. In the current study, we analyzed individuals whose seasonal variations were assessed but who had no diagnosis of mental disorder, and therefore the findings do not disagree. With respect to *ARNTL*, the *TT* genotype of rs2290035 was associated with less seasonal variation in energy level. In our earlier study, it was the heterozygous genotype that was overrepresented among patients with winter depression [16] of which 96% report routine seasonal variation in energy level [48].

As a new finding, we found an association of *CLOCK* rs2412646 with social activity. Support for the role of *CLOCK* in the behavioral activity is provided by a study in which another *CLOCK* variant, rs1801260 (T311C), was linked to the diurnal activity levels in depressed patients [49], [50], and on the other hand in mice disruption of CLOCK contributes to overactive or manic-like behavior [51]. Social withdrawal is a common feature in depression and *CLOCK* gene polymorphisms have been associated with mood disorders [52], [53] even though contradictory studies exist [54]–[56]. In addition, *NPAS2* is a close analog of *CLOCK* and its polymorphisms have earlier been associated with autism [57], a disorder characterized among others with impaired social interaction and communication.

Our study sample derives from the Finnish population, which has reduced genetic and environmental heterogeneity [58]. Moreover, the sample was identified from an epidemiological cohort that is well-characterized for somatic and psychiatric wellbeing allowing detailed phenotype analysis. However, the small sample size for reproductive phenotypes in women, number of SNPs per gene and multiple testing need to be seen as limitations. The most significant association between *ARNTL* rs2278749 and increased risk of miscarriages remains significant after false discovery rate analysis. To assess the significance of the findings replication in other cohorts is needed.

To conclude, we found that genetic variations in *ARNTL* and *NPAS2* genes associated with fertility and seasonality. Our results thus support the previous observation of the role of these genes in seasonal physiology, whereas this is the first time circadian clock related genetic variants are reported to associate with the human fertility.

## Supporting Information

Text S1 Analyzed phenotypes related to mental well-being.
(0.02 MB PDF)

Table S1 SNPs with p-value of less than or equal to 0.01.
(0.02 MB PDF)

Table S2 Haplotypes with p-value of less than 0.05.
(0.01 MB PDF)

Figure S1 LD plots for NPAS2, ARNTL, ARNTL2 and CLOCK.
(0.03 MB PDF)
