# Supplementary material for: ARNTL (BMAL1) and NPAS2 Gene Variants Contribute to Fertility and Seasonality
Source: PLoS One. 2010 Apr 2;5(4):e10007. doi: 10.1371/journal.pone.0010007 (PMC2848852; doi:10.1371/journal.pone.0010007)
Supplement: Text S1 — Analyzed phenotypes related to mental well-being. (0.02 MB PDF) [file pone.0010007.s001.pdf]

Text S1. Analyzed phenotypes related to mental well-being.

The participants completed a modified 21-item Beck Depression Inventory (BDI) [1] as adapted and validated for the Finnish population [2]. The factor matrix was calculated using the maximum likelihood principle, and the standard orthogonal Varimax rotation was computed in order to examine the degree of correlation among factors using the RELIAB module of the Survo MM program, release 2 (www.survo.fi/mm/english.html), producing a two-factor solution: factor one (BDI1) including the items #1 to #12 and #14, and factor two (BDI2) including the items #13, #15 to #19 and #21 to #22.

The health-related quality of life was assessed with the 15-item 15D [3], and mental well-being was assessed with the 12-item General Health Questionnaire (GHQ) [4]. For the 15D, a three-factor solution was produced: factor one (15-D1) including the items of usual activities, mobility, discomfort and symptoms, sexual activity, breathing, vision, elimination, and hearing; factor two (15-D2) including the items of depression, distress, vitality, and sleeping; and factor three (15-D3) including the items of eating, speech, and mental function. For the GHQ, a two factor solution was produced: factor one (GHQ1) including the items #1 to #2, #5 to #9 and #12, and factor two (GHQ2) including the items #3 to #4 and #10 to #11.

The daytime sleepiness was assessed with the 8-item Epworth Sleepiness Scale (ESS) [5]. For the ESS, a two-factor solution was produced: factor one (ESS1) including the items of sensitivity of falling asleep in calm situations, i.e. reading, watching television, sitting quietly, after lunch, as a passenger in a car for an hour without breaks, and afternoon rest, and factor two (ESS2) including the items of sensitivity of falling asleep in interactive situations, i.e. during conversation, and when driving a car and stopping in traffic lights. In addition, the participants filled in how many hours they usually sleep per day (in 24 hours). To assess the intensity of work-related burnout feelings among the employees, the 16-item Maslach Burn Out Inventory was used. For the questionnaires in

detail, see http://www.terveys2000.fi/lomakkeet/en/t2002en.pdf, and for the methods in detail, see http://www.terveys2000.fi/doc/methodologyrep.pdf]

References

1. Beck AT, Ward CH, Mendelson M, Mock J, Erbaugh J. (1961) An inventory for measuring depression. Arch Gen Psychiatry 4: 561-571.

2. Raitasalo R. (2007) Mielialakysely. Suomen oloihin Beckin lyhyen depressiokyselyn pohjalta kehitetty masennusoireilun ja itsetunnon kysely. Helsinki: Kela, Sosiaali- ja terveysturvan tutkimuksia 86.

3. Sintonen H. (2001) The 15D instrument of health-related quality of life: Properties and applications. Ann Med 33(5): 328-336.

4. Goldberg DP, Hillier VF. (1979) A scaled version of the general health questionnaire. Psychol Med 9(1): 139-145.

5. Johns MW. (1991) A new method for measuring daytime sleepiness: The Epworth sleepiness scale. Sleep 14(6): 540-545.
