# Supplementary material for: ARNTL (BMAL1) and NPAS2 Gene Variants Contribute to Fertility and Seasonality
Source: PLoS One. 2010 Apr 2;5(4):e10007. doi: 10.1371/journal.pone.0010007 (PMC2848852; doi:10.1371/journal.pone.0010007)
Supplement: Table S1 — SNPs with p-value of less than or equal to 0.01. (0.02 MB PDF) [file pone.0010007.s002.pdf]

**Table S1. SNPs with p-value≤0.01.**

| Reproduction variables | Gene | SNP | Test | Re | Beta | S.E. | L95% CI | U95% CI | P-value |
|---|---|---|---|---|---|---|---|---|---|
| Number of pregnancies | ARNTL | rs2278749 | REC | *TT* | 7.2 | 2.12 | 3.04 | 11.35 | 0.001 |
| Number of miscarriages | ARNTL | rs2278749 | REC | *TT* | 4.65 | 0.83 | 3.03 | 6.27 | 2.50E-07 |
| Number of miscarriages | NPAS2 | rs11673746 | DOM | *T+* | -0.64 | 0.2 | -1.03 | -0.25 | 0.002 |
| Number of miscarriages | NPAS2 | rs11673746 | ADD | *T* | -0.47 | 0.15 | -0.77 | -0.16 | 0.003 |

| Seasonal variation variables | Gene | SNP | Test | Re | OR/Beta | S.E. | L95% CI | U95% CI | P-value |
|---|---|---|---|---|---|---|---|---|---|
| GSS | NPAS2 | rs2305160 | DOM | *A+* | -0.81 | 0.24 | -1.29 | -0.33 | 0.001 |
| GSS | NPAS2 | rs2305160 | ADD | *A* | -0.57 | 0.19 | -0.94 | -0.2 | 0.003 |
| GSS | ARNTL | rs2290035 | REC | *TT* | -0.92 | 0.31 | -1.53 | -0.3 | 0.004 |
| GSS1 | NPAS2 | rs6725296 | ADD | *A* | 0.27 | 0.08 | 0.11 | 0.42 | 0.001 |
| GSS1 | NPAS2 | rs6725296 | DOM | *A+* | 0.27 | 0.08 | 0.1 | 0.43 | 0.001 |
| GSS2 | NPAS2 | rs2305160 | DOM | *A+* | -0.19 | 0.07 | -0.34 | -0.05 | 0.008 |
| Seasonal variation in weight | NPAS2 | rs6725296 | ADD | *A* | 1.95 | 0.2 | 1.32 | 2.89 | 0.001 |
| Seasonal variation in weight | NPAS2 | rs6725296 | DOM | *A+* | 1.95 | 0.21 | 1.29 | 2.96 | 0.002 |
| Seasonal variation in weight | NPAS2 | rs2305160 | DOM | *A+* | 0.63 | 0.18 | 0.44 | 0.89 | 0.01 |
| Seasonal variation in appetite | ARNTL | rs2290035 | REC | *TT* | 0.47 | 0.26 | 0.28 | 0.78 | 0.004 |
| Seasonal variation in appetite | ARNTL | rs2290035 | ADD | *T* | 0.7 | 0.13 | 0.54 | 0.91 | 0.007 |
| Seasonal variation in energy | ARNTL | rs2290035 | REC | *TT* | 0.45 | 0.24 | 0.28 | 0.72 | 0.001 |
| Vitamin D | NPAS2 | rs6725296 | DOM | *A+* | 4.38 | 1.68 | 1.09 | 7.67 | 0.009 |

| Well-being variables | Gene | SNP | Test | Re | OR/Beta | S.E. | L95% CI | U95% CI | P |
|---|---|---|---|---|---|---|---|---|---|
| ESS2 | NPAS2 | rs2305160 | REC | *AA* | 0.44 | 0.15 | 0.14 | 0.74 | 0.004 |
| ESS2 | CLOCK | rs2412646 | REC | *TT* | 0.42 | 0.16 | 0.11 | 0.73 | 0.009 |
| Social activity | CLOCK | rs2412646 | REC | *TT* | 0.2 | 0.53 | 0.07 | 0.56 | 0.002 |

Results presented for the rare allele/genotype.
Abbreviations: ADD, additive model; DOM, dominant model; REC, recessive model; Re, reference allele / genotype(s); OR/Beta, odds ratio/regression coefficient; S.E., standard error; L/U95% CI, lower/upper bound on confidence interval;GSS, Global seasonality score; GSS1, Global seasonality score factor 1; GSS2, Global seasonality score factor 2;  ESS2, Epworth Sleepiness Scale factor 2.
