# Supplementary material for: ARNTL (BMAL1) and NPAS2 Gene Variants Contribute to Fertility and Seasonality
Source: PLoS One. 2010 Apr 2;5(4):e10007. doi: 10.1371/journal.pone.0010007 (PMC2848852; doi:10.1371/journal.pone.0010007)
Supplement: Table S2 — Haplotypes with p-value of less than 0.05. (0.01 MB PDF) [file pone.0010007.s003.pdf]

Table S2. Haplotype associations, p<0.05.

Variable	Gene	SNP1	SNP2	Haplotype	F	Beta	P-value
15-D1	*ARNTL2*	RS1037921	RS2306074	*CG*	0.08	-0.107	0.041
15-D2	*CLOCK*	RS2412646	RS11240	*TC*	0.28	-0.086	0.046
15-D2	*CLOCK*	RS2412646	RS2412648	*TCT*	0.28	-0.086	0.046
15-D2	*CLOCK*	RS11240	RS3805151	*CTT*	0.28	-0.086	0.046
15-D2	*CLOCK*	RS2412646	RS3805151	*TCTT*	0.28	-0.086	0.046
15-D3	*CLOCK*	RS2412646	RS11240	*CC*	0.39	-0.061	0.019
15-D3	*CLOCK*	RS2412648	RS3805151	*TT*	0.61	0.061	0.019
15-D3	*CLOCK*	RS2412648	RS3805151	*GC*	0.34	-0.058	0.028
15-D3	*CLOCK*	RS11240	RS3805151	*CGC*	0.34	-0.058	0.028
15-D3	*CLOCK*	RS2412646	RS3805151	*CCGC*	0.34	-0.058	0.028
15-D3	*CLOCK*	RS11240	RS2412648	*CG*	0.34	-0.056	0.034
15-D3	*CLOCK*	RS2412646	RS2412648	*CCG*	0.34	-0.056	0.034
Vitamin D	*CLOCK*	RS2412646	RS11240	*CG*	0.34	-2.65	0.017
Vitamin D	*CLOCK*	RS11240	RS2412648	*GT*	0.34	-2.65	0.017
Vitamin D	*CLOCK*	RS2412646	RS2412648	*CGT*	0.34	-2.65	0.017
Vitamin D	*CLOCK*	RS11240	RS3805151	*GTT*	0.34	-2.65	0.017
Vitamin D	*CLOCK*	RS2412646	RS3805151	*CGTT*	0.34	-2.65	0.017

Abbreviations: SNP1, SNP ID of left-most (5') SNP; SNP2, SNP ID of left-most (3') SNP; F, Frequency in sample; Beta, regression coefficient.
