# Supplementary material for: ARNTL (BMAL1) and NPAS2 Gene Variants Contribute to Fertility and Seasonality
Source: PLoS One. 2010 Apr 2;5(4):e10007. doi: 10.1371/journal.pone.0010007 (PMC2848852; doi:10.1371/journal.pone.0010007)
Supplement: Figure S1 — LD plots for NPAS2, ARNTL, ARNTL2 and CLOCK. (0.03 MB PDF) [file pone.0010007.s004.pdf]

Figure S1. LD plots for *NPAS2*, *ARNTL*, *ARNTL2* and *CLOCK*.

NPAS2

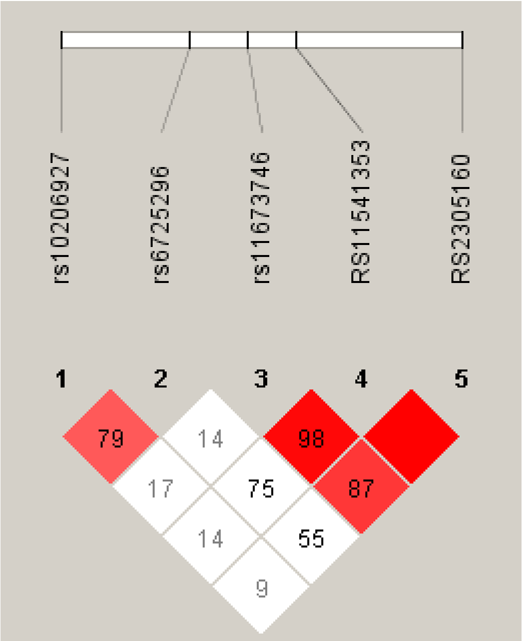

ARNTL2

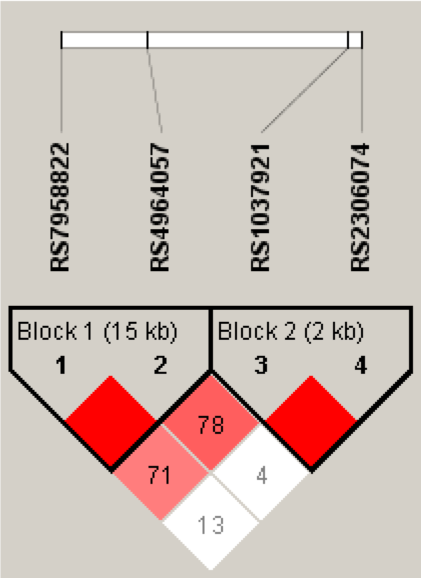

ARNTL

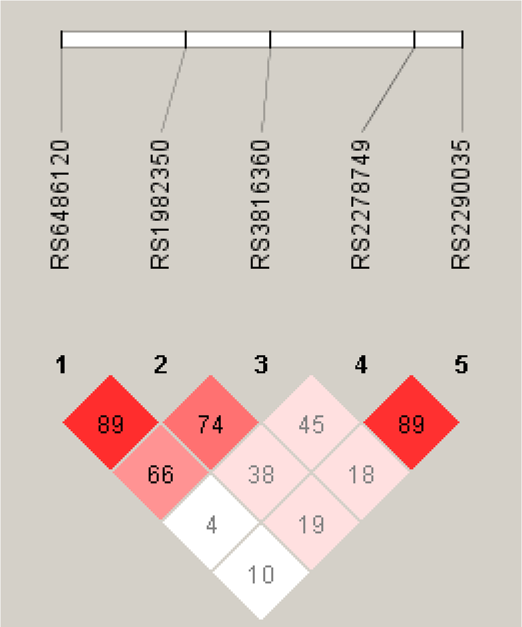

CLOCK

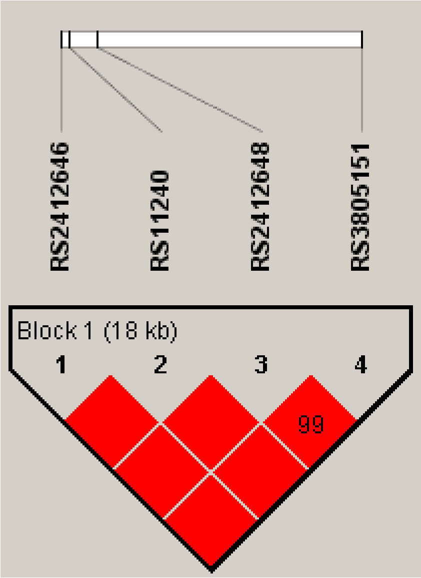

D prime values shown, except if 1.0.
